# Anatomy of the heart with the highest heart rate

**DOI:** 10.1111/joa.13640

**Published:** 2022-02-06

**Authors:** Yun Hee Chang, Boris I. Sheftel, Bjarke Jensen

**Affiliations:** ^1^ Department of Medical Biology University of Amsterdam, Amsterdam, Cardiovascular Sciences, Amsterdam UMC Amsterdam The Netherlands; ^2^ A.N. Severtsov Institute of Ecology and Evolution RAS (Russian Academy of Sciences) Moscow Russian Federation

**Keywords:** cardiac conduction system, development, evolution, pulmonary veins, trabeculation

## Abstract

Shrews occupy the lower extreme of the seven orders of magnitude mammals range in size. Their hearts are large relative to body weight and heart rate can exceed a thousand beats a minute. It is not known whether traits typical of mammal hearts scale to these extremes. We assessed the heart of three species of shrew (genus *Sorex*) following the sequential segmental analysis developed for human hearts. Using micro‐computed tomography, we describe the overall structure and find, in agreement with previous studies, a large and elongate ventricle. The atrial and ventricular septums and the atrioventricular (AV) and arterial valves are typically mammalian. The ventricular walls comprise mostly compact myocardium and especially the right ventricle has few trabeculations on the luminal side. A developmental process of compaction is thought to reduce trabeculations in mammals, but in embryonic shrews the volume of trabeculations increase for every gestational stage, only slower than the compact volume. By expression of Hcn4, we identify a sinus node and an AV conduction axis which is continuous with the ventricular septal crest. Outstanding traits include pulmonary venous sleeve myocardium that reaches farther into the lungs than in any other mammals. Typical proportions of coronary arteries‐to‐aorta do not scale and the shrew coronary arteries are proportionally enormous, presumably to avoid the high resistance to blood flow of narrow vessels. In conclusion, most cardiac traits do scale to the miniscule shrews. The shrew heart, nevertheless, stands out by its relative size, elongation, proportionally large coronary vessels, and extent of pulmonary venous myocardium.

## Graphical abstract

Chang et al. investigate the hearts of shrews, the mammals with the greatest heart rate. Mostly, the hearts have the expected anatomical traits, but the extent of the myocardium in the pulmonary veins is more extreme than in any other mammal.

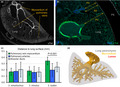

## 1 INTRODUCTION

Shrews are eutherian insectivore mammals that can be tiny. Adult Eurasian least shrew (*Sorex minutissimus*) and Eurasian pygmy shrews (*Sorex minutus*), which we study in this report, only weigh a few grams. They occupy therefore the lower extreme of the seven orders of magnitude that mammals range in size. Because not much is known of their cardiac anatomy (Rowlatt, 1990; Vornanen, 1989), it is not clear whether typical traits of mammal hearts scale to such miniscule sizes. Valves and chamber wall thicknesses would be predicted to scale linearly to cavity size according to the law of Laplace (Jensen, 2021; Seymour & Blaylock, 2000) and typical proportions of valves, walls, and cavities might well scale to shrews. In contrast, resistance to blood flow is inversely related to vessel diameter raised to the power of four according to the Hagen‐Poiseuille equation and very small arteries and veins in the shrew may impose high resistance.

Blood perfusion constantly must be critical in shrews since with the tiny body size also comes with the highest rates of mass‐specific metabolism among mammals. This metabolism is supported by the greatest mass‐specific cardiac outputs, or volume of blood per gram tissue pumped per minute (Jurgens et al., 1996; Morrison et al., 1959). Whereas in human, it takes 1 min to circulate the entire blood volume, in a shrew it takes a few seconds (Schmidt‐Nielsen, 1984). A key component of the prodigious circulation is the incredibly fast heart rate, which may exceed a thousand beats a minute in the smaller shrew species (Jurgens et al., 1996; Morrison et al., 1959; Nagel, 1986; Vornanen, 1992). In many mammals, a well‐developed cardiac conduction system initiates the cardiac impulse in the sinus node. While fast activation in the atriums is ongoing, activation is slowly propagated through the atrioventricular (AV) node and then rapidly spread throughout the ventricles via the His bundle, bundle branches and Purkinje fibers (Davies, 1942; Dobrzynski et al., 2013). Because shrews have extremely high heart rates, their conduction system may be extensive but this has not been investigated. Besides high heart rates, an additional component in achieving great cardiac output is likely a relatively large stroke volume because relative heart mass is substantially greater in shrews than in most other mammals (Bartels et al., 1979; Pucek, 1965; Vornanen, 1989).

Besides the role of scale on the structure of the shrew heart, phylogeny likely has an impact as well. Some traits of mammal hearts are universally shared such as two atriums and two ventricles (Rowlatt, 1990). Also, the valves of the aorta and the pulmonary artery always have three leaflets each and the left AV valve, or the mitral valve, always has two leaflets. Between mammals variation does exist, however, and the right AV valve can be dominated by a single large leaflet, have two leaflets, or have three leaflets such as in human. Other examples of variation is the number of caval veins connecting to the right atrium (RA) which is either two or three and the number of pulmonary veins connecting to the left atrium (LA) which varies even more (Kroneman et al., 2019). In short, across mammals, most structures exhibit some degree of variation, but it is not known where shrew hearts fall on such spectrums of variation. There is no consensus on how to analyze the gross anatomy of a mammal heart albeit a common approach is to describe structures in the same order as blood flows through the heart (Marais & Crole, 2021; Kareinen et al., 2020). One similarly ordered and broadly used approach for human hearts is the sequential segmental analysis which is sufficiently versatile to be applicable to crocodile hearts (Cook et al., 2017). Here we followed this manner of analysis.

Our specimens were caught in the wild in fall traps. By coincidence, three of the trapped shrews were pregnant females. From the embryos of these, we could study the development of the ventricular walls. Early cardiogenesis has been described for the house shrew and demonstrates the presence of highly trabeculated ventricles (Yasui, 1992; Yasui, 1993), while anatomical studies on adult animals of the closely related moles of the genus *Talpa* suggest the adult ventricle may have few trabeculations only (Rowlatt, 1990). Gestational changes to the extent of ventricular trabeculations have attracted much attention in the context of so‐called “noncompaction” cardiomyopathy (Chin et al., 1990; Del Monte‐Nieto et al., 2018; D'Silva & Jensen, 2020). Focus has been on a process of “compaction”, whereby trabeculated muscle on the luminal side of the ventricle is added to the compact wall (Sedmera et al., 2000). There is very little quantitative evidence, however, for such a process (Faber, D'Silva, et al., 2021; Rychterova, 1971). Instead, there is much stronger quantitative support for the trabecular and compact layers can grow at different rates and when they do, it changes the proportion of trabecular‐to‐compact myocardium (Blausen et al., 1990; Faber, Hagoort, et al., 2021; Faber, Wüst, et al., 2021). If the ventricular walls of adult shrews have few trabeculations, while the embryonic walls are much trabeculated, shrews may be a good model system to test whether compaction or differential growth rates better characterize gestational changes to the trabeculated layer.

The primary research question of this study is whether typically traits of mammal hearts scale to the miniscule size of shrews.

## 2 MATERIAL AND METHODS

### 2.1 Animals

All animals were collected from near Yenisei Ecological Station of A.N. Severtsov Institute of Ecology and Evolution RAS, Turukhansk district of Krasnoyarsk region (N62°17′, E89°02′). The collection of animals occurred between 21st and 30th of August 2018 in compliance with the guidelines of the A.N. Severtsov Institute of Ecology and Evolution RAS and the guidelines of the American Society of Mammalogists (Sikes & The Animal Care and Use Committee of the American Society of Mammalogists, 2016). Briefly, animals were collected from fall traps filled with water containing alcohol and then deskinned and fixed in either 10% or 20% formalin for one day and then kept in 70% ethanol until further use. Of the Eurasian pygmy shrew (*S minutus*), we used six heart‐lung preparations for the description of the formed heart and four embryos from each of three pregnant females for the description of developing hearts (Table 1). The embryos were at three different times of gestation, which when staged on heart morphology, we assessed to correspond to mouse heart development ages embryonic day 10.5, 12.5, and 16.5. The actual gestational ages was probably a few days older since our youngest stage (10.5) had more developed limb buds than the oldest stages described by Yasui (Yasui, 1992; Yasui, 1993), which were approximately 12 days old. Besides specimens of the Eurasian pygmy shrew, we also used heart‐lung preparations of formed animals of two Eurasian least shrew (*S minutissimus*) and two taiga shrew (*Sorex isodon*; Table 1). No embryos were found for these species. The animals of the three species were either adult (overwintered 2017–2018 and sexually mature), juvenile (from 2018 and not sexually mature), or, in one case, subadult (from 2018, but sexually mature). The age and sexual maturity was based on wear of teeth and fur, and state of internal organs (Zaitsev et al., 2014).

**TABLE 1:** Overview of the used specimens. #XXX is the specimen‐specific code

Specimen	Body mass (g)	Micro‐CT	IHC	Picro‐sirius red
*Sorex minutus*				
#124 juvenile female	3,3		Yes	Yes
#127 adult male	4,6	Yes	Yes	Yes
#250 adult male	4,9			Yes
#261 juvenile female	3,3		Yes	Yes
#286 juvenile male	3,0		Yes	
#630 adult female	6,1	Yes		
Embryos				
#699 E10.5 (n = 4)		yes (n = 1)	yes (n = 1)	yes (n = 2)
#422 E12.5 (n = 4)		yes (n = 1)	yes (n = 1)	yes (n = 2)
#630 E16.5 (n = 4)		yes (n = 1)	yes (n = 1)	yes (n = 2)
*Sorex minutissimus*				
#368 subadult female	2,3			Yes
#639 juvenile female	2,0		Yes	Yes
*Sorex isodon*				
#394 juvenile female	9,3	Yes		
#395 juvenile male	9,7			Yes
#396 juvenile female	8,6		Yes	

Abbreviation: Exx.x, embryonic day xx.x (based on likeness to mouse).

### 2.2 Micro‐computed tomography

The three heart‐lung specimens that were investigated with micro‐CT, two *S minutus* and one *S isodon*, were first stained for 2 days with Lugol's solution (1.75 g I_2_ and 2.50 g KI, both from Fischer Scientific, dissolved in 100 ml deionized water; Metscher, 2009). The volume of Lugol's solution was at least 10 times that of the tissue and the solution was kept in the dark during the staining. The specimens were then immobilized by imbedding in an agar solution (1.3 g per 100 ml water). Subsequently, they were scanned at isotropic 10 μm resolution using a Bruker, Skyscan 1272 (Blom et al., 2019). All shown images of micro‐CT are of specimen 127 which had the best tissue‐lumen contrast of the two *S minutus* specimens.

### 2.3 Sectioning and histology

The hearts that were investigated with histology were embedded in paraplast and cut in 10 or 12 μm sections in either the transverse or frontal plane (four‐chamber view). Staining was with saturated picro‐sirius red in which muscle becomes orange and collagen becomes red following 2 min differentiation in 0.01 M HCl (Jensen et al., 2017). Imaging of the stained slides was done with a Leica DM5000 light microscope.

### 2.4 Immunohistochemistry

With fluorescent immunohistochemistry, as done previously (Jensen et al., 2017), we detected smooth muscle actin (SMA), cardiac troponin I (cTnI) and funny current channel, or hyperpolarization‐activated cyclic nucleotide‐gated potassium channel 4 (Hcn4), as well as nuclei using DAPI (dilution 1:1000, D9542; Sigma‐Aldrich). For SMA, we used the primary antibody RRID:AB_476701 (Sigma‐Aldrich, dilution 1:400) which was visualized by a fluorescently labeled secondary donkey anti‐mouse antibody coupled to Alexa 555 (dilution 1:250; Thermo Fisher Scientific, RRID:AB_2536180). For cTnI, we used the primary antibody RRID:AB_154084 (Hytest, dilution 1:300) which was visualized by a fluorescently labeled secondary donkey anti‐goat antibody coupled to Alexa 488 (dilution 1:250; Thermo Fisher Scientific, RRID:AB_2762838). For Hcn4, we used the primary antibody RRID:AB_2120042 (Millipore, dilution 1:200) which was visualized by a fluorescently labeled secondary donkey anti‐rabbit antibody coupled to Alexa 647 (dilution 1:250; Thermo Fisher Scientific, RRID:AB_2536183). In addition, for identification of the sinus node we tested Isl1 (RRID:AB_2126323) and Shox2 (RRID:AB_945451) but we never detected positive nuclei. We did not seek to clarify whether the absence of signal was due to the absence of the protein or deterioration of the epitopes due to suboptimal tissue fixation and preservation. Slides were viewed and photographed with a Leica DM6000B fluorescent microscope. For estimations of the volume of trabecular and compact myocardium in embryos, we stained for cTnI as above on one section of 10 μm thickness per 100 μm (nine sections for specimen 699; eight sections for specimen 422; 14 sections for specimen 630).

### 2.5 Analyses and statistics

We imported to Amira (version 2020.2, ThermoFisher Scientific) the micro‐CT image series (three adult heart‐lung specimens, one embryo of each of the three gestational ages) and the histology image series of three embryos of each of the three gestational ages and the images used for estimation of ventricular trabecular and compact tissue volume. Concerning *S minutissimus*, from the histological section series of the entire adult heart‐lung preparation we imported to Amira every 25th section, in total 13 sections for specimen 368 and 14 sections for specimen 639. Structures of interest were then labelled and their volume was derived using the “Materials Statistics” tool. To measure distances of structures of the lungs, we imported images to ImageJ (Abràmoff et al., 2004; version IJ 1.46r) and used the straight line selection tool. We used a one‐way ANOVA to test for differences in distance to the lung surface of pulmonary venous myocardium, pulmonary arteries, and alveolar ducts.

## 3 RESULTS

### 3.1 Heart position and orientation

**FIGURE 1:**
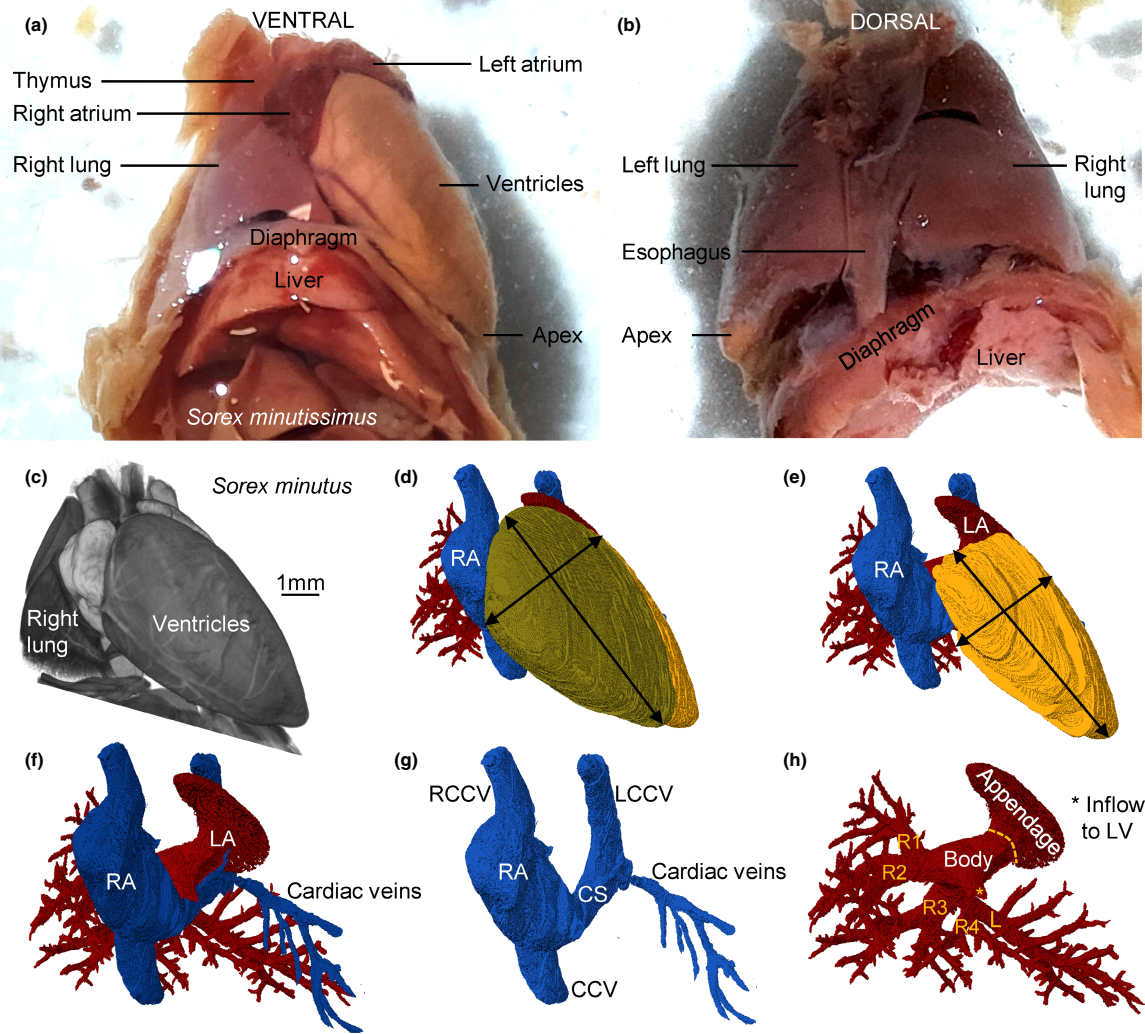
Topology of the shrew heart. (a, b) Macroscopic photos of the ventral (a) and dorsal (b) view of the heart of *Sorex minutissimus* (specimen 637). Notice the caudal‐left position of the apex and the great elongation of the ventricle. (c) Volume rendering of micro‐CT of *Sorex minutus* (specimen 127), showing the heart in its approximately attitudinally correct position when viewed ventrally. (d) Three‐dimensional reconstruction based on the micro‐CT of (c), showing the right atrium (RA) and right ventricle (RV) are the most ventral chambers. (e) The left ventricle is very elongate. (f, g) Three caval veins connect to the RA. (h) The left atrium (LA) has a large appendage. It receives one stem into which drains veins from the first two lobes of the right lung (R1–R2) and a second stem into which drains veins from the last two lobes of the right lung (R3–R4) and the left lung (l). CCV, caudal caval vein; CS, coronary sinus; L(R)CCV, left (right) cranial caval vein

The body mass differed between the three investigated species (Table 1) and so did the size of the heart. The hearts were proportionally large, their tissue volume comprised more than 0.7% of fresh body mass (Table 2) and this percentage would likely have been greater still if fresh hearts had been measured (Vornanen, 1989). Besides the difference in absolute size of the heart between the species, there were no gross morphological differences between the hearts. The position of the shrew heart is much like in human (Figure 1). Within the thoracic cavity, the heart is mostly on the left side, and immediately caudal to the heart is the diaphragm. The ventricles in particular are quite elongate and the rather pointy apex is made up of the apex of the left ventricle (LV) (Figure 1a–d). There is no interventricular sulcus and the border between the left and right ventricle (RV) is subtle. The left atrium (LA) is the most cranial and dorsal chamber, the right atrium (RA) is the right‐most chamber, and the right ventricle (RV) rests on the diaphragm and is the most caudal and ventral chamber (Figure 1c). The LV is longer and narrower than the RV, with the length/breadth index of the left and RV being 1.5 and 1.3, respectively (Figure 1d,e). Three caval veins connect to the RA (Figure 1f,g). Two pulmonary veins connect to the LA (Figure 1f,h).

**TABLE 2:** Volume of the heart and the pulmonary venous myocardium

Specimen	Body mass (g)	Cardiac volume (mm^3^)	Cardiac index (%)	PVM volume (mm^3^)	PVM/cardiac volume (%)
*Sorex minitus*					
286	3.0	23.34	0.78	0.488	2.09
261	3.9	28.42	0.73	0.360	1.27
127	4.0	35.70	0.78	1.163	3.26
*Sorex minutissimus*					
368	2.3	16.02	0.70		
639	2.0	15.92	0.80		

Abbreviation: PVM, pulmonary venous myocardium.

### 3.2 The right side of the heart

#### 3.2.1 Caval veins

**FIGURE 2:**
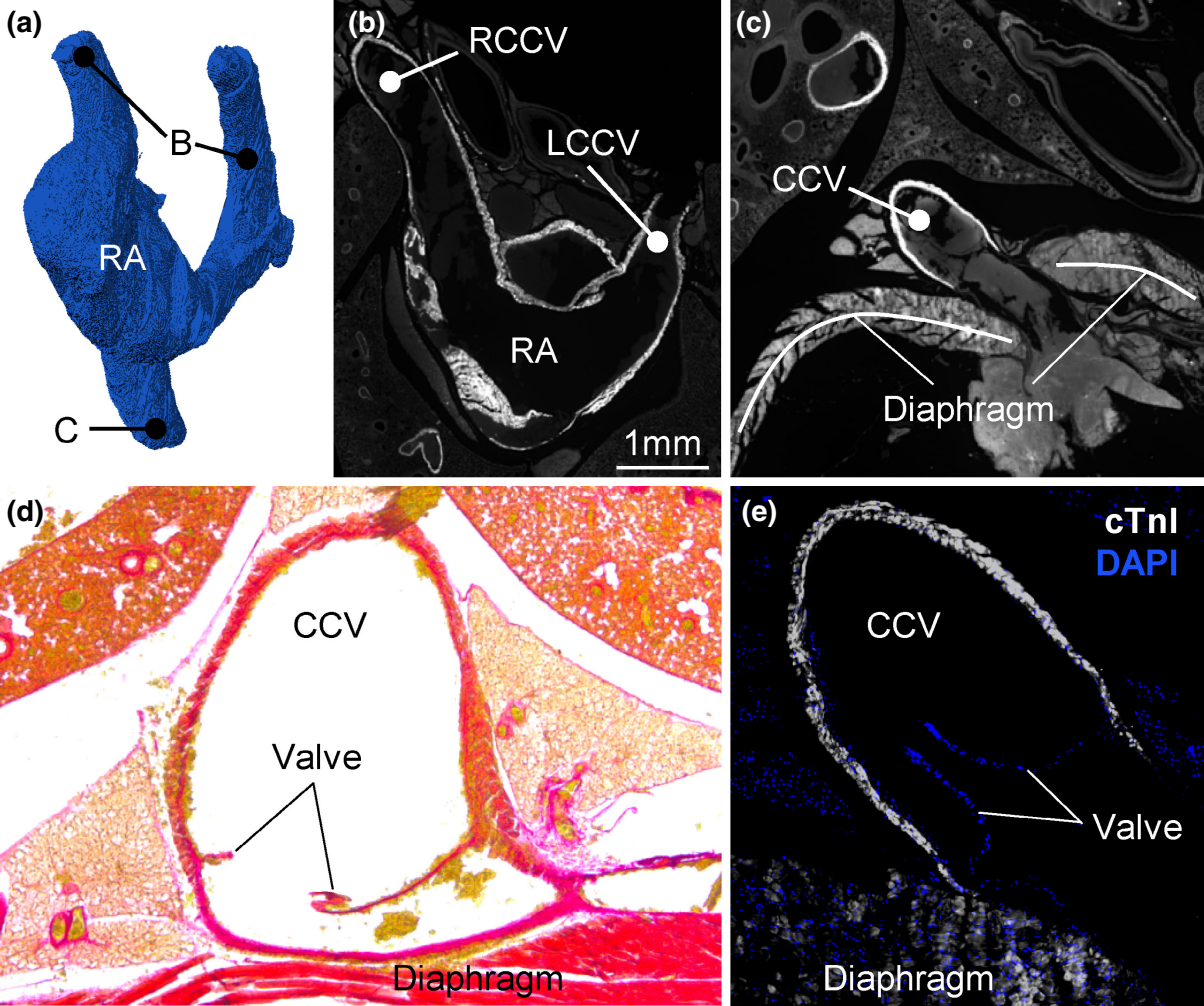
Extensive myocardial sleeves of the caval veins. (a) Three‐dimensional reconstruction based on the micro‐CT of the lumen of the right atrium (RA) and caval veins. Label B points to the approximate cranial‐most extent of the myocardial sleeves which is documented in image (b). Label C points to the caudal‐most extent of the myocardial sleeves which is documented in image (c). (b) Immunohistochemical detection of cTnI in the left and right cranial caval vein (LCCV and RCCV respectively). (c) Immunohistochemical detection of cTnI in the caudal caval vein (CCV). (d) Picro‐sirius red staining of histological section, showing the venous valve at the myocardial‐venous boundary in *Sorex minutissimus* (for the sake of presentation, blood inside the vein has been painted over with white). (e) Immunohistochemical detection of cTnI and nuclei (DAPI), showing the venous valve in the CCV at the myocardial‐venous boundary in *Sorex minutus*. All sections are in the horizontal plane

Three caval veins connect to the RA, namely the caudal caval vein (CCV), right cranial caval vein (RCCV), and the left cranial caval vein (LCCV) (Figure 1f–g). The part of the LCCV that is most proximal to the RA could be considered the equivalent of the coronary sinus of the human heart as it receives the great cardiac veins, it lies in the left AV groove and opens into the vestibule of the RA (Figure 1g). There is no bridging vein between the RCCV and the LCCV, while such vein can be found in human with persistent left superior caval vein (Kula et al., 2011). All three caval veins have myocardium extending to the pericardial reflection (Figure 2a–c), whereas in human there is hardly any myocardium in the inferior caval vein (Noheria et al., 2013). The distal‐most parts of the cranial veins where often not included in the excised preparations, but when they were we found venous valves cranial to the myocardial‐venous boundary. Also, at the myocardial‐venous boundary of the CCV we found a valve in all three species (Figure 2d,e).

#### 3.2.2 The RA and atrial septum

**FIGURE 3:**
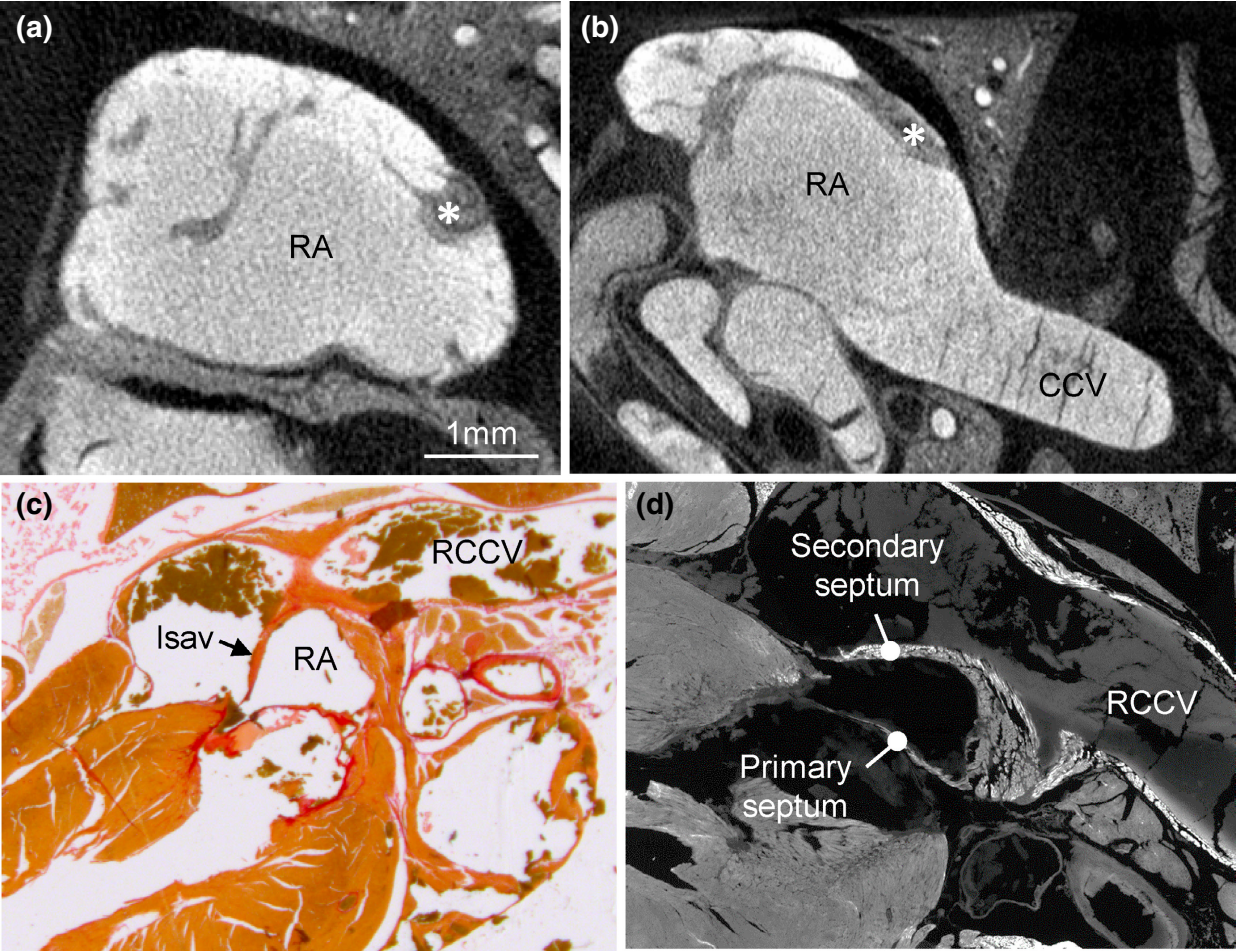
Anatomy of the right atrium. (a) Micro‐CT image showing the wall of the right atrium (RA) comprises a meshwork of trabeculations, the greatest among which is the crista terminalis (asterisk). (b) There is a comparatively extensive smooth‐walled part, or body. (c) Histological section stained with picro‐sirius red showing a well‐developed left leaflet of the sinuatrial valve (lsav) that extends from the junction of the right cranial caval vein (RCCV) to the base of the atrial septum. (d) Immunohistochemical detection of cardiac troponin I showing the atrial septum has a primary component that is thin and contains much non‐myocardial tissue and a secondary component mostly of myocardium. All sections are approximately in the horizontal plane. CCV, caudal caval vein

A very prominent pillar‐shaped muscular ridge, the crista terminalis is found in the dorsolateral wall of the RA, where it divides the atrium into a dorsal smooth portion, or body, and a lateral trabeculated portion, or appendage (Figure 3a). The trabeculations are thin compared to those of the LA (see below) and, when compared to the human RA, the trabeculations are configured more as a network than parallel pectinate muscles. Also, in contrast to human, the body is bigger than the appendage (Figure 3b). The trabeculation extends to the area immediately around the right AV valve and atrial septum, except for the area of the vestibule. There is a well‐developed left leaflet of the sinuatrial valve, whereas the right leaflet is much less prominent (Figure 3c). The atrial septum comprises a thick secondary atrial septum composed of predominantly myocardium and a thin primary septum, or flap valve, composed of some myocardium besides endocardium and connective tissue (Figure 3d). The cranial aspect of the secondary septum, which in human is essentially a fold (Anderson et al., 2014), in the shrew also takes the appearance of a folding‐in of the atrial roof albeit the sulcus is traversed by bits of myocardium.

#### 3.2.3 The RV and pulmonary artery

**FIGURE 4:**
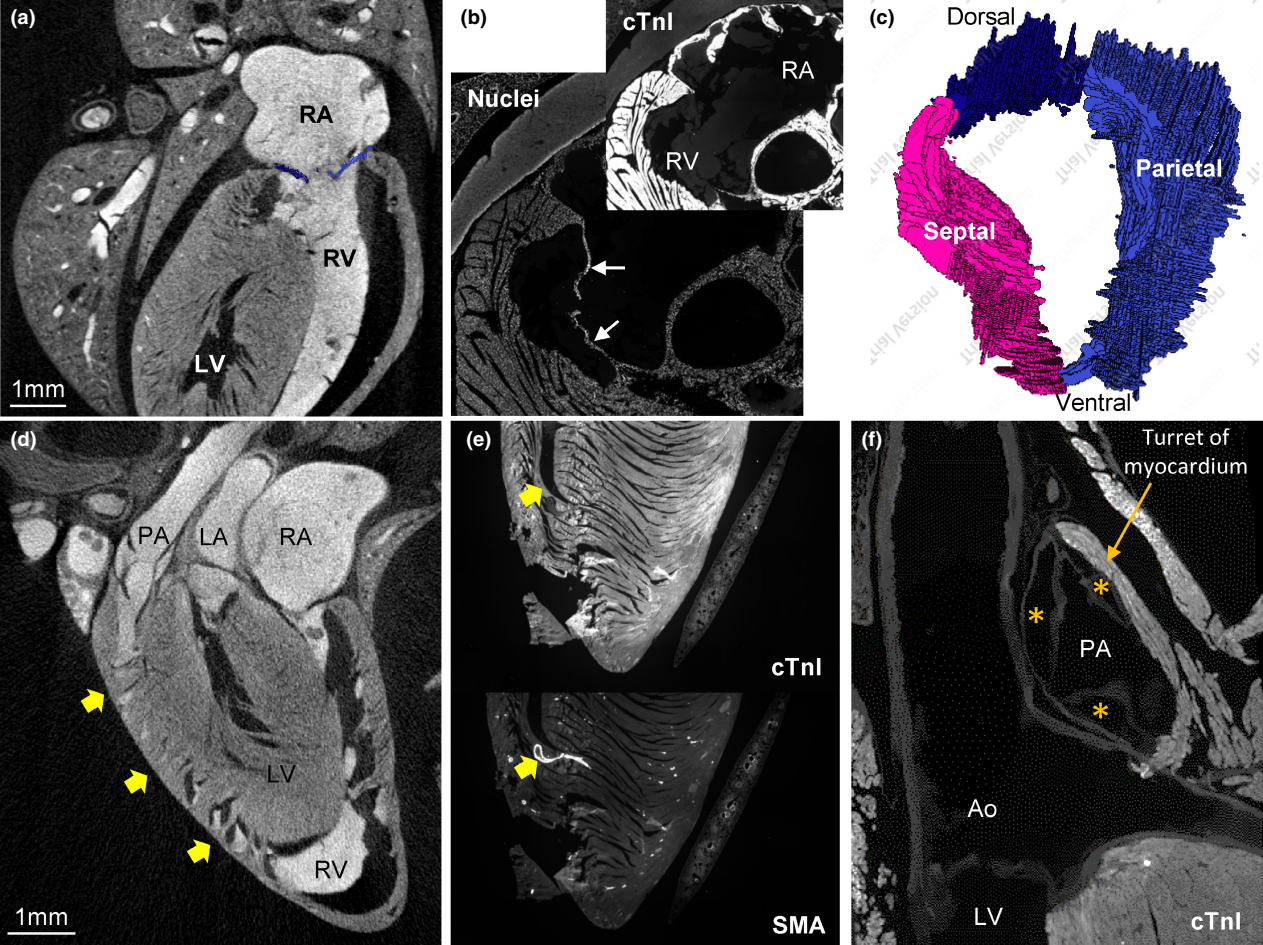
Anatomy of the right ventricle. (a) Micro‐CT image showing that the right ventricle (RV) wraps around the left ventricle (LV). Notice the smooth appearance of the septal surface and parietal wall, including an absence of prominent papillary muscles. (b) Immunohistochemical detection of cardiac troponin I and nuclei (DAPI) showing the atrioventricular valve is membranous (white arrows) and without myocardium. (c) Reconstruction of the atrioventricular valve, showing a prominent septal leaflet and a parietal leaflet that has a cleft such that it is divided into two parts. (d) Micro‐CT image showing the boundary between wall and septum contains numerous trabeculations (arrows). (e) Immunohistochemical detection of cardiac troponin I and smooth muscle action (SMA) showing the trabecula septo‐marginalis and moderator band (arrow), within which there is a large coronary artery (its wall contains SMA). (f) Immunohistochemical detection of cardiac troponin I showing the valve of the pulmonary artery (PA) has three leaflets that hinge in a “turret” of myocardium. All sections are approximately in the horizontal plane

The RV looks to wrap around the septal part of the LV (Figure 4a) as is typical in mammals. The AV valve is membranous and of connective tissue (Figure 4b). It comprises a large septal leaflet and a large parietal leaflet (Figure 4c). A prominent cleft subdivides the parietal leaflet in a smaller dorsal part and a large ventral part (Figure 4c). The septal surface has a mostly smooth surface. Consequently, the appearance of the papillary muscles and chordae tendineae is very subtle (Figure 4a). Most of the luminal side of the ventricular wall in fact has a smooth, or a‐trabecular, appearance including the outflow tract. At the transition from septum to wall, however, there are well‐developed trabeculations (Figure 4d). Also, a fairly prominent trabecula septo‐marginalis was found in the typical mammalian position (ventro‐apically) and it hosted a proportionally quite large coronary artery (Figure 4e). There are three cusps to the pulmonary arterial valve. As in human, these hinge in ventricular myocardium that forms a myocardial turret around the base of the pulmonary artery (Figure 4f).

**FIGURE 5:**
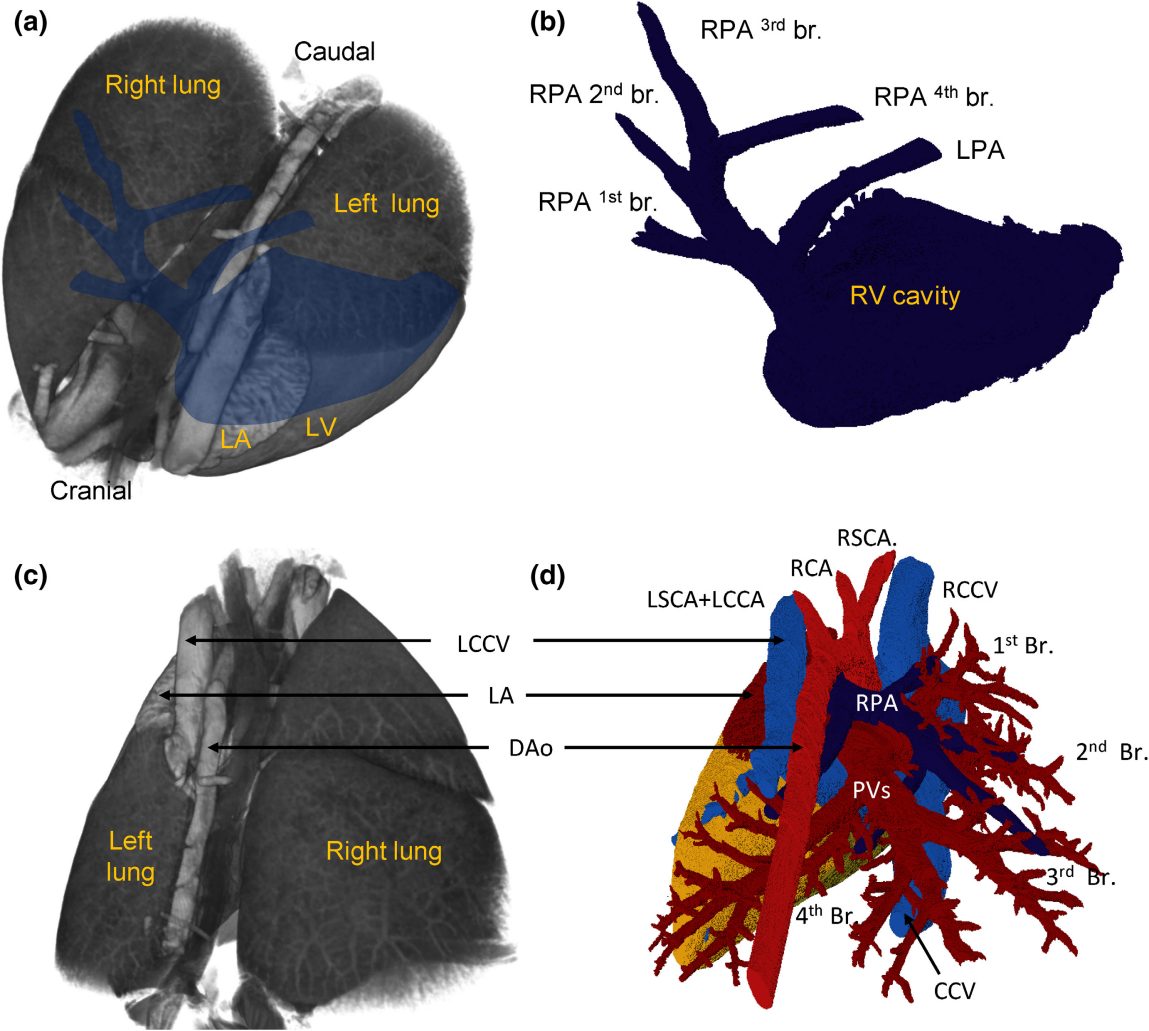
Pulmonary artery. (a) Volume rendering of micro‐CT with reconstructed right ventricle and main branches of the pulmonary artery (blue silhouette). (b) Virtual lumen cast of the cavity of the right ventricle (RV) and the pulmonary artery showing the four main branches to the right pulmonary artery (RPA first–fourth) and the solitary left pulmonary artery (LPA). (c, d) Dorsal view of the pulmonary circulation

The main trunk of the pulmonary artery is short and it bifurcates into one branch to each lung (Figure 5). Just as the right lung is bigger than the left, the right branch of the pulmonary artery is bigger than the left. The right branch lies between the ascending and descending aorta and splits into four branches, one branch for each of the four lobes of the right lung (Figure 5a,b). The smaller solitary left pulmonary artery has an angle of approximately 75° to the main trunk and goes into the left lung which has one lobe only (Figure 5c,d).

#### 3.2.4 The ventricular septal structures

**FIGURE 6:**
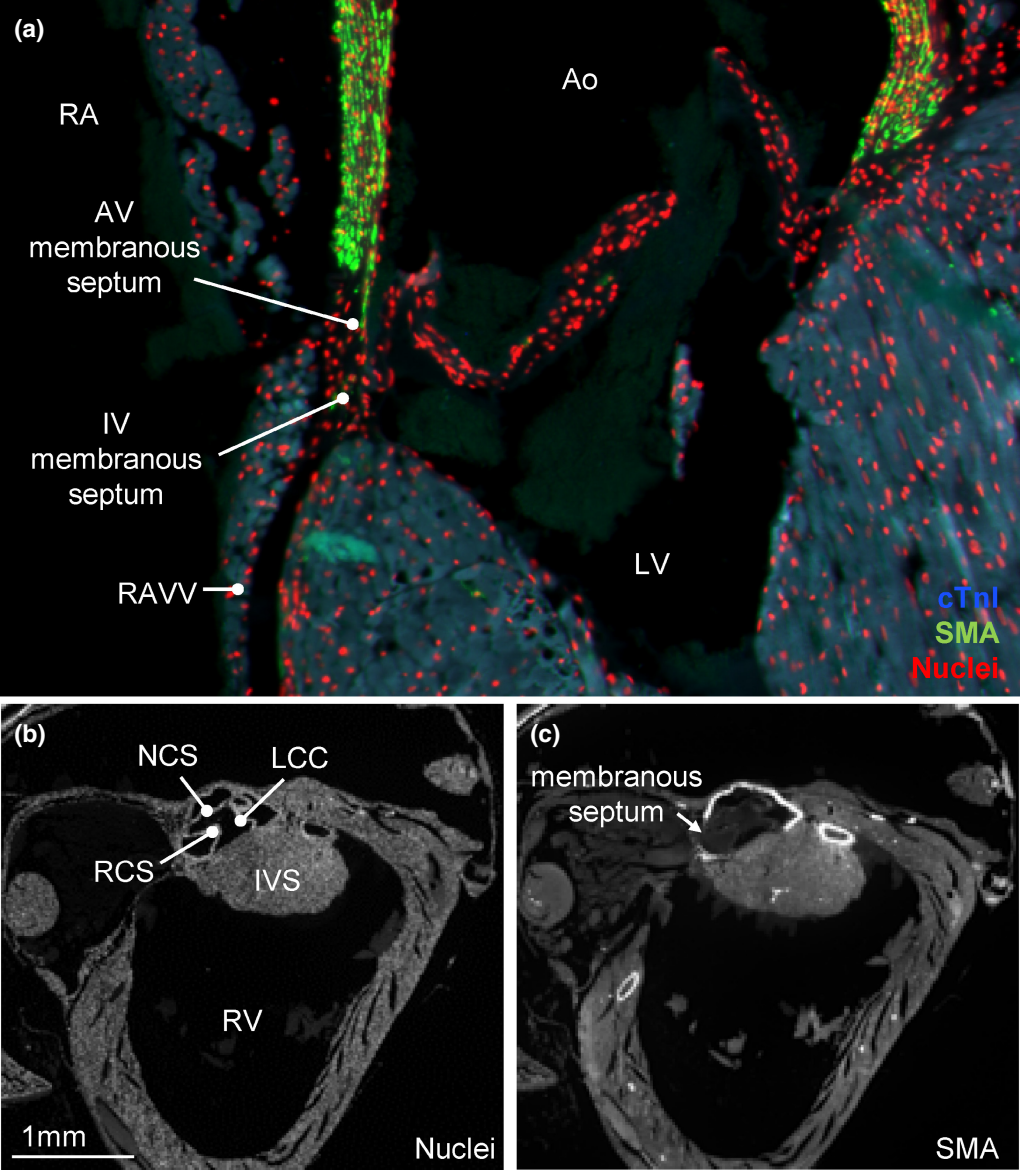
Membranous septum. (a) Immunohistochemistry showing the small membranous septum (cTnI negative) connects with the septal leaflet of the right atrioventricular valve (RAVV). Above this hinge line is the atrioventricular (AV) membranous septum and below it is the interventricular (IV) membranous septum. (b, c) The atrioventricular membranous septum is below the base of the non‐coronary cusp (NCC) and right coronary cusp (RCC) of the aortic valve. All sections are approximately in the horizontal plane. Ao, aorta; LCC, left coronary cusp; LV, left ventricle; RV, right ventricle

A small membranous septum was found below between the base of the aorta and the crest of the myocardial ventricular septum. The right AV valve hinges onto the membranous septum and thereby divides into an AV component (it separates the cavities of the RA and the LV) and an interventricular component (Figure 6a). A substantially offset in the hinge‐line for the tricuspid valve relative to the mitral valve hinge‐line was not seen. The AV membranous septum occupies the base of the gap between the non‐coronary and right coronary cusps of the aortic valve (Figure 6b,c).

#### 3.2.5 Pulmonary veins

The left‐sided atrial body receives two very short stems of pulmonary veins, with the right stem coming from the first two lobes of the right lung and the left stem coming from the third and fourth lobe of the right lung together with the solitary vein of the left lung (Figure 1f). The right stem passes between the entrance of the RCCV and CCV and left stem passes over the coronary sinus to reach the LA.

**FIGURE 7:**
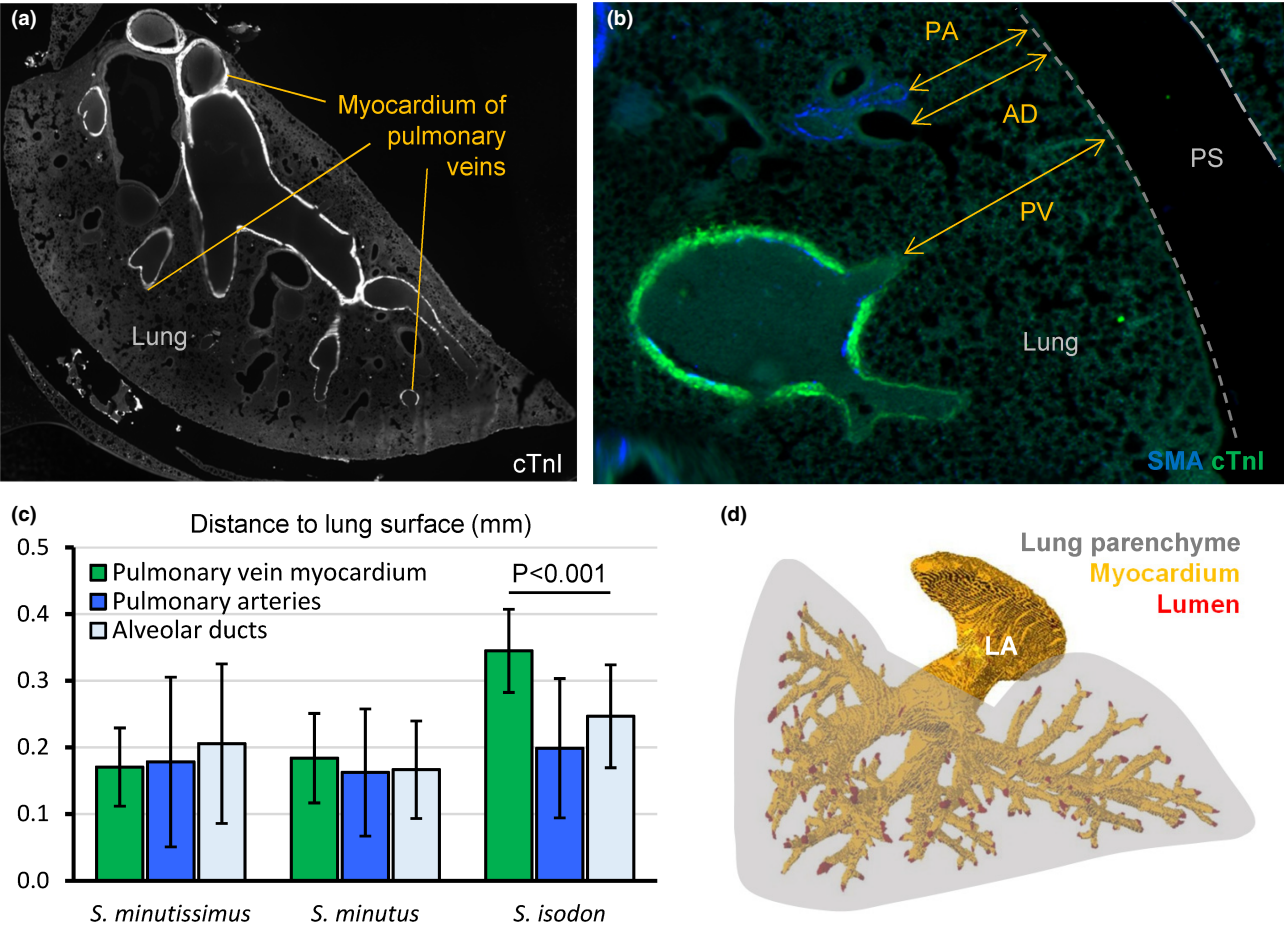
Extreme extent of pulmonary venous myocardial sleeves. (a) Myocardium in the walls of the pulmonary veins reached the farthest parts of the lungs (immunohistochemistry of lung of *Sorex minutus*). (b) Detailed view showing the pulmonary veins (PV), alveolar ducts (AD) and pulmonary arteries (PA) all extended to the proximity of the lung surface. (c) Pulmonary venous myocardium extended as close to the lung surface as the terminal parts of the alveolar ducts and the pulmonary arteries in *Sorex minutissimus* (one‐way ANOVA, *p* = 0.106; one specimen, 15 sections, 27 measurements per structure) and *S. minutus* (one‐way ANOVA, *p* = 0.573; three specimens, four or five sections per specimen, 53 measurements per structure), but not in *Sorex isodon* (one‐way ANOVA, *p* < 0.001; one specimen, 10 sections, 39 measurements per structure). (d) Reconstruction in ventral view of the pulmonary venous lumen (red) and myocardial sleeves of *S. minutus*, illustrating that only the distal most parts of the pulmonary veins were without sleeves. Both sections are approximately in the horizontal plane. PS, pleural space

A remarkable feature of the shrew heart is the extent of the myocardial sleeves of the pulmonary veins (Figure 7). In *S minutissimus* and *S minutus* these sleeves reach within 0.2 mm of the lung surface, even in the distal parts of the lungs (Figure 7c,d). Measured as the distance to the lung surface, the pulmonary venous myocardium extends as far as the pulmonary arteries and the terminal parts of the alveolar ducts (Figure 7c,d). Despite the great extent of the pulmonary venous myocardium, it only constitutes approximately 2% of total myocardial volume (Table 2). In *S isodon* the sleeves were also extensive, but they did not extend as far as the pulmonary arteries and the terminal parts of the alveolar ducts (Figure 7c).

#### 3.2.6 Left atrium

**FIGURE 8:**
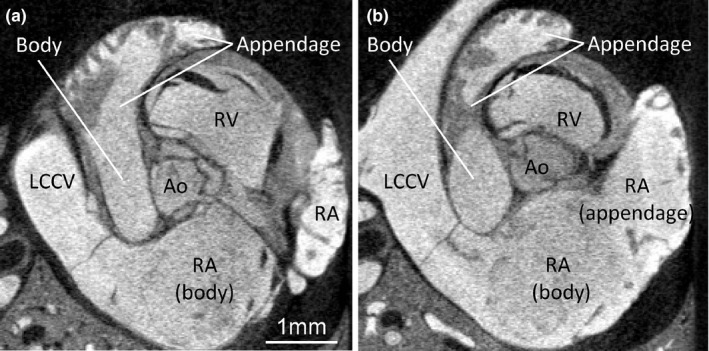
Left atrium. (a) Image from micro‐CT showing the left atrium has a smooth‐walled body and a proportionally large trabeculated appendage. (b) Trabeculations of the left atrium are more coarse than those of the right atrium (RA). Ao, aorta; LCCV, left cranial caval vein; RV, right ventricle. Both images are approximately in the transverse plane

The LA is situated dorso‐cranially. It has a venous component (body), a trabeculated component (appendage), and a vestibule (Figure 8). The body has the two orifices of the pulmonary veins. The appendage is proportionally large when compared to the human setting. It is clog‐shaped and its junction with the body is relatively narrow (Figure 1f). The pectinate muscles are much more extensive in the left atrial appendage than the right atrial appendage (Figure 8). Even though the LA lacks the muscular bundle like crista terminalis in the RA, the junction between the appendage and body is well defined by the coarse trabeculation in the appendage (Figure 8).

#### 3.2.7 Left ventricle and aorta

**FIGURE 9:**
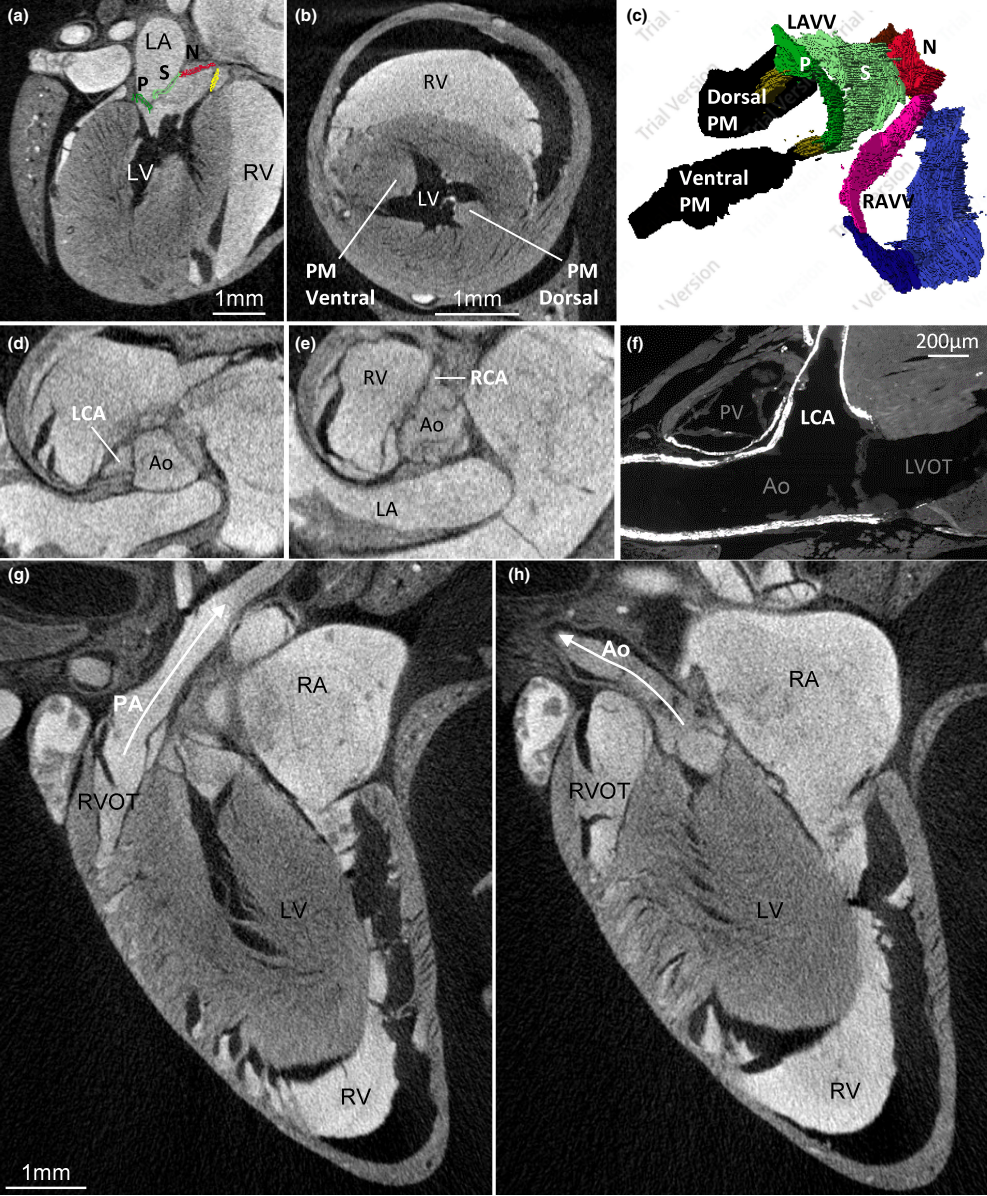
Left ventricle and aorta. (a) Image in the horizontal plane from micro‐CT showing the left atrioventricular valve has a parietal leaflet (P) and a septal leaflet (S) which is continuous with the aortic leaflet of the non‐coronary sinus (N). (b) Image in the transverse plane showing there are two large papillary muscles (PM). (c) Reconstruction in dorsal view of the left atrioventricular valve (LAVV) and the right atrioventricular valve (RAVV) and their tension apparatus, the latter of which is much more developed in the LV than the RV. (d, e) Images in the transverse plane showing the origins of the left (LCA) and right coronary artery stem (RCA) within the sinuses of the aortic valve. (f) Immunohistochemical of detection of smooth muscle actin on a section in the horizontal plane showing the LCA is proportionally very large, its diameter is almost a half of the diameter of the aorta (Ao) and the base of the pulmonary arterial valve (PV). (g, h) Dorsal view of image in the horizontal plane from micro‐CT showing showing the aorta and the pulmonary artery are oriented at very different angles as is typical of mammals. L(R)A, left (right) atrium; L(R)VOT, left (right) ventricular outflow tract; L(R)V, left (right) ventricle

The left AV valve has two leaflets and two papillary muscles (Figure 9). From the valve margins, chordae tendineae connect to the ventral papillary muscle which emerges from the ventricular septum and to the dorsal papillary muscle that emerges from the ventricular free wall (Figure 9b,c). The papillary muscles and tension apparatus of the LV are much more prominent than those of the RV. With the exception of the septo‐parietal trabeculation of the RV, the trabeculations of the LV are coarser than those of the RV, and this setting therefore resembles that of the pig but not that of human where the RV has the coarser trabeculations (Crick et al., 1998). The left ventricular outflow tract is without trabeculations. The septal AV leaflet and fibrous continuity are the part of the outflow tract (Figures 6a and 9a).

There are three cusps to the aortic valve and each of the two coronary arteries arise from its own aortic sinus as in human (Figure 9d,e). The left coronary artery has a proportionally very large diameter, approximately 1:2 when compared to that of the aorta, and this ratio in human would be much closer to 1:10 (Figure 9f). Its actual diameter is only approximately 200 μm, however, which is the diameter of an arteriole. The right coronary artery is almost as large as the left. Both coronary arteries immediately become intramural in their course through the ventricular mass and their main stems are in the parietal walls rather than at the boundary of the left and RV. Consequently, the ventricular surface does not have large coronary vessels and an interventricular sulcus that demarcate the left and RV as, for example, in human and pig (Crick et al., 1998).

The aorta and the pulmonary trunk have a spiral relationship (Figure 9g,h). The aortic arch crosses cranially to the bifurcation of the pulmonary arteries (Figure 5d). The ascending aorta is located to the right of the trunk of the pulmonary artery. The aortic arch gives rise to only two branches leading cranially. The descending aorta lies between the esophagus and LCCV (Figure 5d).

#### 3.2.8 Development of ventricular trabeculation

**FIGURE 10:**
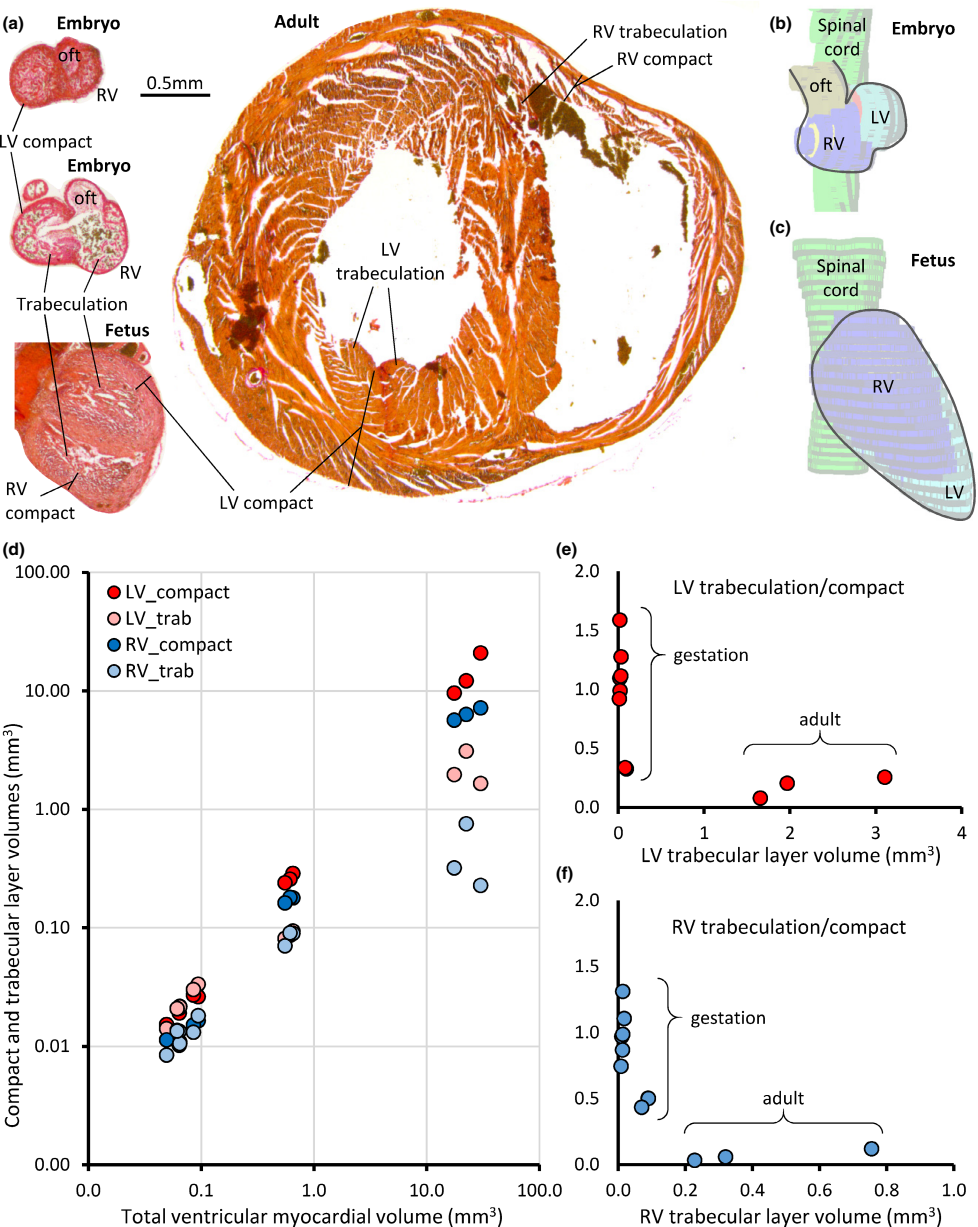
Development of ventricular trabeculation. (a) Transverse histological sections stained with picro‐sirius red of the ventricles of the four stages of ontogeny that were investigated (on the same scale). The ventricular lumen of the embryonic and fetal ventricles are proportionally more trabeculated than the adult heart. (b, c) Reconstructions based on histological sections. The spinal cord (green) indicates the body long‐axis. Notice the elongate shape of the fetal ventricle. (d) The four layers comprising the total myocardial ventricle, were all significantly linearly correlated to the total volume (LV compact, *p* < 0.001; LV trabeculation, *p* < 0.001; RV compact, *p* < 0.001; RV trabeculation, *p* = 0.004). Nonetheless, the trabecular layers had a much smaller volume than the compact layers in the adult heart. (e, f) The proportion of trabecular myocardium volume to compact myocardium volume was significantly negatively correlated to trabecular layer volume (LV trabeculation, *p* = 0.024; RV trabeculation, *p* = 0.002). LV, left ventricle; LV trab, left ventricle trabeculation; oft, outflow tract; RV, right ventricle; RV trab, right ventricle trabeculation

From embryonic development to adulthood, the heart undergoes a great enlargement (Figure 10a). This includes the acquisition of a pronounced elongate shape already in intrauterine development (Figure 10b,c). The ventricles of the adult heart are characterized by having little trabeculated myocardium in proportion to compact myocardium (Figure 10a). This suggests extensive compaction has taken place, where compaction can be defined as a reduction of the trabecular layer by the addition of trabeculations to the compact wall (Faber, D'Silva, et al., 2021). To detect compaction, we investigated the gestational change to the volumes of the trabecular and compact layers of both ventricles, in hearts that by outward appearance resembled hearts of mouse from gestational ages E10.5, E12.5, and E16.5 (De Boer et al., 2012). All myocardial layers were positively and significantly correlated to the total myocardial volume of the ventricles (Figure 10d; Pearson's linear correlations, *p* < 0.01 for each layer). The compact layers increased more in volume than did the trabeculated layers. Ontogeny, therefore, associated with a proportional decrease in trabecular muscle driven by greater growth of compact muscle rather than an absolute decrease in trabeculation as would be predicted if compaction occurred (Figure 10e,f).

#### 3.2.9 The cardiac conduction system

**FIGURE 11:**
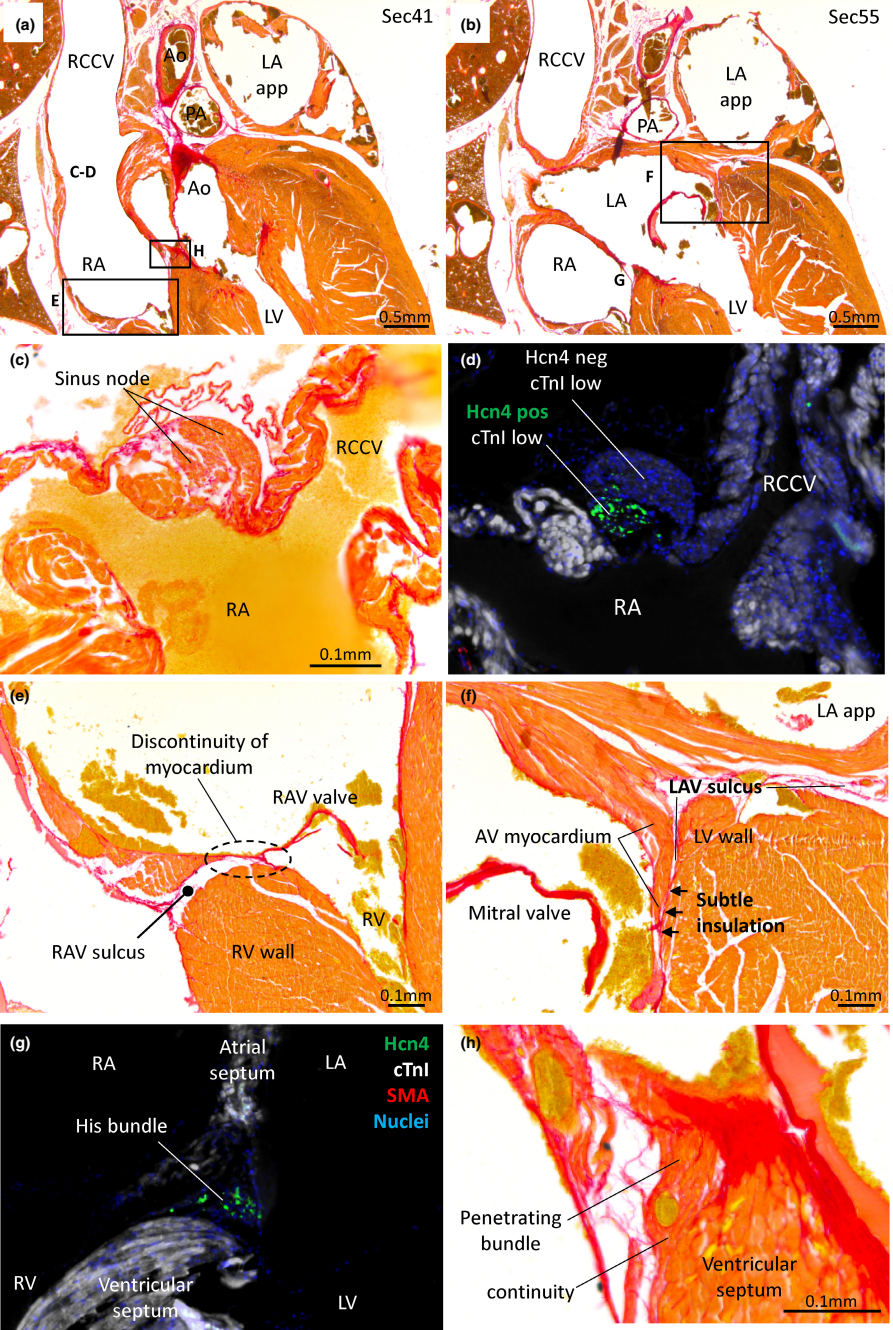
Cardiac conduction system. (a) Histological section stained with picro‐sirius red giving an overview of the sinuatrial (c, d) and right atrioventricular junction (e). (b) Overview of the left atrioventricular junction (f), 280 μm dorsal to image (a). (c, d) Sinus node on the junction of the right cranial caval vein (RCCV) and right atrium (RA), characterized by greater collagen infiltration (c) and expression of Hcn4 while having weak expression of cTnI (d, immunohistochemistry). (e) Histological section stained with picro‐sirius red showing the right atrioventricular junction has no myocardial connection between the atrium and ventricle (RV) despite the junction contains very little fibro‐fatty tissue. (f) The left atrioventricular junction, showing only a subtle amount of insulation between the atrioventricular (AV) and ventricular (LV) myocardium. (g) Immunohistochemistry of the crest of the ventricular septum showing the His bundle (Hcn4 expression, weak cTnI expression) localized in a position much like the one indicated with ‘G’ in image (b). (h) The His bundle penetrates its insulation and becomes continuous with the ventricular septum. This image is a zoom‐in of the region indicated with ‘H’ in image A. All sections are approximately in the horizontal plane. Ao, aorta; LA app, left atrial appendage; L(R)AV, left (right) atrioventricular; PA, pulmonary artery

Given the extremely high heart rates of shrews, it could be presumed that their hearts would contain a structurally pronounced conduction system. To investigate whether this was the case, we surveyed three histological series (Figure 11a,b). A sinus node was found in the parietal and ventral junction of the RCCV and the RA (Figure 11c). It was identifiable by being node‐like in appearance, by being relatively rich in collagen and by a low expression of cardiac troponin I (Figure 11d). In addition, Hcn4 which is a key marker of the conduction system (Boyett et al., 2021), was expressed in a subset of the myocardium with a low expression of troponin I (Figure 11d) which is another characteristic of conduction tissue (Sizarov et al., 2011). This presumed sinus node was small, approximately 100 μm wide and 500 μm dorso‐ventrally long in all three investigated specimens. Concerning the AV conduction axis, an insulating plane disrupted the AV myocardial continuity in the right and left AV junction (Figure 11e,f). At the base of the atrial septum, there was myocardium which was relatively dispersed by collagen and which expressed Hcn4 and expressed relatively little troponin I (Figure 11g). This myocardium therefore had the appearance of the penetrating bundle of His. Ventrally, this myocardium came into continuity with the myocardium of the ventricular septal crest (Figure 11h). We did not identify structures that resembled the AV node or bundle branches, but many of the sections that could have contained these structures also had artefacts from blood and connective tissue that had come loose during the staining procedures.

## 4 DISCUSSION

The shrew heart is in most ways a typical mammal heart, despite being at the extreme lower end of the seven orders of magnitude that mammals range in size. Its exceptional traits can be summarized as its large relative size and great elongation, which have been reported before (Vornanen, 1989), and, as we show here, the extreme extent of the pulmonary venous myocardium. Sleeves of myocardium around the pulmonary veins are found in many mammals including human (Rowlatt, 1990). The sleeves are typical confined to the parts that are most proximal to the LA and they are thought to act as throttle valves of pulmonary venous return (Nathan & Gloobe, 1970). In the shrew, however, there is almost no part of the pulmonary venous tree that is without a myocardial sleeve. It is therefore difficult to envision the role of throttle valve to this myocardium. In human, ectopic pacing of the atria often originates from the pulmonary venous myocardium and this setting requires pulmonary vein isolation by catheter‐ablation. While electrocardiograms have been reported for shrews (Jurgens et al., 1996; Morrison et al., 1959; Nagel, 1986; Vornanen, 1989), it is not known whether their extensive pulmonary venous myocardium associates with a propensity to develop atrial arrhythmias.

### 4.1 Differences between species of shrew

The primary aim of this study was to identify the traits that species of shrew share and how these relate to the same traits in other mammals. Our data also reveal, however, some degree of difference between the three investigated species. First, the absolute size of heart is, unsurprisingly, greater in the heavier species, *S isodon*. Second, in *S isodon* the pulmonary venous myocardium appears to extend less far into the lungs than the pulmonary artery, whereas in *S minutus* and *S minutissimus* the extent of the two structures was not different. The observations on the lung tissues, however, is based on few specimens only and had the study included more specimens of *S isodon* and *S minutissimus* we may have been able to detect more differences. Overall, the hearts of the investigated species appear highly similar, even in their highly unusual traits such as the proportional size and elongation of the heart.

### 4.2 Traits that shrews share with most mammals

Shrews and most mammals have three caval veins. The human setting of having a regressed left caval vein is less common (Carmona et al., 2018; Jensen, Boukens, et al., 2014; Rowlatt, 1990). Compared to human, the caval vein myocardium is extensive, but many mammals have similarly extensive myocardium, that is, the myocardium extends to the vicinity of the pericardial border (Jensen, Boukens, et al., 2014; Nathan & Gloobe, 1970). The number of pulmonary veins that connect to the LA is more variable in mammals than in other tetrapods (Kroneman et al., 2019) and two veins, as in shrews, is not uncommon (Rowlatt, 1968). The AV valves and tricuspid pulmonary and aortic valves of the shrews were typical of mammals (Rowlatt, 1990). In monotreme and marsupial mammals, the right AV valve can be dominated by the parietal leaflet (Lankester, 1882; Runciman et al., 1992), but in the shrews the septal leaflet was well‐developed as is common in eutherian mammals (Rowlatt, 1990). Eutherians are distinct from other mammals by having a second atrial septum which leaves a circular depression called the oval fossa on the right face of the atrial septum (Jensen et al., 2019; Röse, 1890; Rowlatt, 1990). The shrews also have an oval fossa and its dorso‐cranial rim is provided by a fold in the atrial roof in a manner that much resembles the human setting (Anderson et al., 2014).

Given the extremely high heart rates of shrews, one could presume an unusual cardiac conduction system. The cardiac conduction system has been identified on the basis of histological characters such as weak stain and richness in collagen (Davies, 1942; Ho et al., 2003) and on the presence of the funny current channel, Hcn4 (Boyett et al., 2021; Sizarov et al., 2011). We show in the shrew the presence of myocardium that is insulated by collagen and that expresses Hcn4 where a mammal sinus node and His bundle would be expected. A lot of our histology was perturbed by loose and displaced connective tissue and blood, and this hampered the identification of the AV conduction axis in particular. We therefore suggest that the absence of a clear identification of an AV node and bundle branches in our data should not be seen as strong evidence for the absence of these structures. From the data we have, we consider it unlikely that the shrew cardiac conduction system is extensive.

### 4.3 Unusual traits of the shrew heart

A right ventricular wall and septum almost free of trabeculations is not typical of mammals but it is found in shrews, as we report here, and bats, squirrels, and mustelids (Rowlatt, 1990). In many ungulates, for example, the LV can have few trabeculations only (Jensen et al., 2016; Rowlatt, 1990) and the shrew LV is also somewhat sparse in trabeculation. Across vertebrates it is always so that the ventricles of embryos are highly trabeculated (Jensen et al., 2016), which has also been documented in shrews (Yasui, 1993) and we confirm here. So a proportional change must take place from the embryonic and highly trabeculated setting to the adult setting. Two different processes have been proposed to explain such proportional change. One is that the trabecular and compact layers grow throughout development, but the growth rate of the two layers may differ periodically which then changes the layer proportions (Faber, Wüst, et al., 2021). In agreement with this view, our data show the compact layer has a greater rate of growth than the trabecular layer and this reduces the proportion of trabecular muscle during gestation. The other proposed process is compaction, whereby trabeculations are removed from the trabecular layer and added to the compact wall (Chin et al., 1990; Rychterova, 1971). No decrement of the trabecular layer thickness has been documented, however (Faber, D'Silva, et al., 2021), even though this is a predicted outcome if compaction takes place. In this light, the shrew ventricles are an interesting test for the hypothesis of compaction, because their right ventricular wall is comparatively very smooth. The developmental data, however, do not support a role of compaction but it does support a role of differential growth rates.

### 4.4 Unusual traits that may be ascribed to small size

One unusual trait of the shrew hearts was the little amount of fibro‐fatty tissue that comprised the insulating plane between the atria and ventricles. In the left AV junction, the insulation was so meagre that it was difficult to assess whether the atrial and ventricular myocardium was in fact insulated from each other. Irrespective of the extent of insulation by fibro‐fatty tissues, myocardium that slowly propagates the electrical impulse normally occupies the AV junction and this provides a degree of AV insultion (Aanhaanen et al., 2011; Rentschler et al., 2011). While the relative importance of connective tissue and myocardium with slow propagation cannot be deduced from histology, a functional insulation is present since shrews exhibit a delay between atrial and ventricular activation (Jurgens et al., 1996; Morrison et al., 1959; Nagel, 1986; Vornanen, 1992). In larger animals, millimeters of connective tissue separate the atrial and ventricular myocardium (Ho et al., 2003).

A second unusual trait was the extremely large size of the main branches of the coronary vessels, when these were seen in proportion to the diameter of the aorta and thickness of the ventricular wall. In absolute size, the diameter of the coronary vessels was small and it was within the range of arterioles. In large conduit vessels such as the human aorta, diameter has almost no impact on resistance to blood flow as given by the Hagen‐Poiseuille equation, whereas in the size range of arterioles, diameter change has a very large impact on resistance. In this light, the proportions of aorta‐to‐coronary‐arteries found in human, for example, may not scale to the small size of shrews, because it would reduce the diameter of the coronary arteries so much as to render them high‐resistance vessels.

### 4.5 Highly unusual traits of the shrew heart

A very elongate ventricle is a highly unusual trait in a mammal and it is found in shrews (Vornanen, 1989) and the closely related moles of the genus *Talpa* (Rowlatt, 1968). Otherwise, elongate ventricles appear restricted to animals that are highly elongate themselves, such as snakes and caecilian amphibians (de Bakker et al., 2015; Jensen, Moorman, et al., 2014; Ramaswami, 1944). Shrews and moles are not particularly elongate mammals and the significance of their highly unusual heart shape is not clear. Heart shape may affect function according to the law of Laplace (Vornanen, 1989), but it is not implausible that heart shape is also an outcome of structural restrictions offered by the ribcage, diaphragm and maybe even the lungs. The early developing hearts that were investigated here, and previously (Yasui, 1993), were not much different in shape from developing mouse hearts, whereas the hearts in late gestation were essentially shaped like the adult heart. It is then likely that the shrew heart grows into its unusually long shape, rather than it first acquires a typical mammal fetal shape and then remodels.

Perhaps the most unusual morphological trait of the shrew heart is the extent of the pulmonary venous myocardium. It was previously demonstrated that pulmonary venous myocardium is present in shrews (Endo et al., 1997) and in mice, for example, myocardial sleeves extend three bifurcations up the pulmonary venous tree (Mommersteeg et al., 2007). To the best of our knowledge, however, our report is the first documentation of the extreme extent of the shrew pulmonary venous myocardium which much exceeds that of mouse. The extreme extent aside, the amount of muscle around the pulmonary veins is not much, it is just a few percent of the total cardiac mass. Therefore, even if cardiac muscle is energetically very demanding (Mootha et al., 1997), the pulmonary venous myocardium may not necessarily impose a large metabolic cost and its advantage to organismal performance may not have to be great.

Observations on valves in veins have a long history (Franklin, 1927), but to the best of our knowledge this is the first report of a valve on the myocardial‐venous boundary in the CCV. Whales have a sphincter at the same position (Lillie et al., 2018), but not a valve of leaflets. Even in reptiles, where the caval vein myocardium functions as a chamber and a valve in the CCV would seem advantageous (Jensen et al., 2017; Joyce et al., 2020), there is no valve. Because such valve is rarely sought after and it may easily be obscured by coagulated blood or by its collapse against the vessel wall, a dedicated investigation may be required to establish whether a valve in the CCV is highly unusual among vertebrates.

That the pulmonary and systemic veins contains much myocardium is consistent with them functioning as contractile chambers. The presence of the valve in CCV is also consistent with the intrapericardial caval veins functioning as a chamber, as the valve likely prevents regurgitation during contraction. In reptiles, the caval vein myocardium is electrically activated well before the atria and this aids the filling of the RA (Jensen et al., 2017). If conditions are favorable, the electrical activation of the caval vein myocardium can be detected on the body‐surface electrocardiogram as a unique deflection, the SV‐wave, and which occurs before the P‐wave which heralds the activation of the atria (Jensen et al., 2017; Mullen, 1967; Valentinuzzi & Hoff, 1972). While a P‐wave has been detected on electrocardiograms of shrews (Jurgens et al., 1996; Morrison et al., 1959; Nagel, 1986; Vornanen, 1992), no study has reported a SV‐wave. This is not necessarily evidence against the venous myocardium of shrews being activated before the atrial muscle, since the elicited potentials are likely very small and only a small fraction of electrocardiograms of reptiles, for example, exhibit a SV‐wave (Boukens et al., 2019; Mullen, 1967). Possibly, the systemic and pulmonary venous myocardium in the shrews function as chambers that aid the filling of the right and LA respectively, but we consider it more likely that this myocardium is activated and contracts together with the myocardium of the atria as in other mammals (Jensen, Boukens, et al., 2014).

## 5 CONCLUSION

In this study, we investigated whether typically traits of mammal hearts scale to the extremely small size of shrews and key anatomical structures, such as valves and septums, do scale. Traits that do not scale may be the proportionally very large coronary arteries and the AV junction insulation, which comprise very little tissue. The pronounced elongation of the ventricle, the extreme extent of the pulmonary venous myocardial sleeves, and the valve in the CCV may set shrew hearts aside from other mammal hearts.

## AUTHOR CONTRIBUTION

*Conceptualization of the study*: Bjarke Jensen; *acquisition of data and critical revision of the manuscript*: Yun Hee Chang, Boris I. Sheftel, Bjarke Jensen; *data analysis and interpretation, and drafting of the manuscript*: Yun Hee Chang and Bjarke Jensen.

## Data Availability

The data that support the findings of this study are available from the corresponding author upon reasonable request.
